# Supplementary material for: A Double-Blind, Randomized Intervention Study on the Effect of a Whey Protein Concentrate on E. coli-Induced Diarrhea in a Human Infection Model
Source: Nutrients. 2022 Mar 12;14(6):1204. doi: 10.3390/nu14061204 (PMC8948686; doi:10.3390/nu14061204)
Supplement: Supplementary file 1 [file nutrients-14-01204-s001.zip › nutrients-1575579-supplementary.pdf]

nutrients

MDPI

Supplementary Material

Table S1 Study Schedule

Study days	Pre-screening	1-10	11	12	13	14	15	16	17	18	19-26	27	28
Informed consent and screening													
Restricted intake specific													
Restricted alcohol intake													
Consumption study product													
Standardized evening meal													
Overnight fast													
Infection attenuated E. coli													
Data Safety Monitoring Board											d21		
Collection 24h fecal samples				*	*							*	*
Collection blood sample													
Bristol stool scale, stool frequency and GSRS (online).													
Restricted dairy intake & dietary guidelines (incl. antibiotics)													
Registration medication intake & compliance (online)													

*Fecal samples were collected on day 12 or 13 and on day 27 or 28, since not all subjects have daily bowel movements before and late after *E. coli* inoculation.

Data S1. In- and Exclusion criteria

Inclusion criteria

In order to be eligible to participate in this study, a subject must meet all of the following criteria:

Substantial:

1. Male

2. Age between 18 and 55 years.

3. BMI ≥18.5 and ≤30.0 kg/m2.

4. Healthy as assessed by the NIZO health questionnaire.

Procedural:

5. Ability to follow Dutch verbal and written instructions.

6. Availability of internet connection.

7. Signed informed consent.

8. Willing to accept disclosure of the financial benefit of participation in the study to the authorities concerned.

9. Willing to accept use of all encoded data, including publication, and the confidential use and storage of all data for at least 15 years.

10. Willing to comply with study procedures, including collection of stool and blood samples.

11. Willingness to abstain from high calcium containing products during the study.
12. Willingness to abstain from alcoholic beverages three days before, during and for 4 days after diarrheagenic E. coli challenge.
13. Willingness to abstain from medications that contain acetaminophen, aspirin, ibuprofen, and other nonsteroidal anti-inflammatory drugs, (OTC) antacids and antimotility agents (eg, loperamide) on the three days before, during and for 4 days after diarrheagenic E. coli challenge.
14. Willingness to abstain from probiotics and prebiotics/fibers starting from run-in and during the entire study.
15. Willingness to give up blood donation starting at run-in and during the entire study.

Exclusion criteria

A potential subject who meets any of the following criteria will be excluded from participation in this study:

1. Acute gastroenteritis in the 2 months prior to inclusion.
2. Any confirmed or suspected immunosuppressive or immunodeficient condition including human immunodeficiency virus infection (HIV).
3. Disease of the GI tract, liver, bile bladder, kidney, thyroid gland (self-reported), except for appendicitis.
4. History of microbiologically confirmed ETEC or cholera infection within 3 years prior to inclusion.
5. Symptoms consistent with Travelers' Diarrhea concurrent with travel to countries where ETEC infection is endemic (most of the developing world) within 3 years prior to inclusion, OR planned travel to endemic countries during the length of the study.
6. Vaccination for, or ingestion of cholera within 3 years prior to inclusion, including studies at NIZO.
7. Occupation involving handling of ETEC or Vibrio cholerae currently, or within 3 years prior to inclusion.
8. Vaccination for, or ingestion of ETEC or E coli heat labile toxin, including E. coli challenge studies at NIZO.
9. Evidence of current excessive alcohol consumption (>4 consumptions/day or >20 consumptions/week) or drug (ab)use, and not willing/able to stop this during the study.
10. Known allergy to the following antibiotics: ciprofloxacin, trimethoprim, sulfamethoxazole, and penicillins.
11. Reported average stool frequency of >3 per day or <1 per 2 days.
12. Use of antibiotics (up till 6 months prior to inclusion), norit, laxatives, cholestyramine, antacids H2 receptor antagonists or proton pump inhibitors (during 3 months prior to inclusion).
13. Use of immunosuppressive drugs (e.g. cyclosporine, azathioprine, systemic corticosteroids, antibodies).
14. Vegans.
15. Mental status that is incompatible with the proper conduct of the study.
16. A self-reported milk allergy, lactose intolerance or sensitivity to dairy ingredients.

Procedural:

17. Not having a general practitioner, not allowing disclosure of participation to the general practitioner or not allow to inform the general practitioner about abnormal results.
18. Participation in any clinical trial including blood sampling and/or administration of substances starting 1 month prior to study start and during the entire study.
19. Personnel of NIZO or FrieslandCampina, their partner and their first and second degree relatives.

Fecal organic acid analysis by HPLC

For organic acid analysis, 250 mg of homogenized fecal sample was diluted with 1 ml of 1M perchloric acid (HClO4) to release the organic acids. Lipids and proteins in the fecal sample were removed by centrifugation for 5 min at 20,000 g. Organic acids lactate, acetate, propionate, butyrate, isobutyrate, isovalerate and valerate were determined by high-performance anion-exchange chromatography with UV and refractive index detection. 25 µl of the supernatant was injected on a guard column in series with 2 Rezex ROA-Organic Acid H+ Analytical Columns (Phenomenex, Torrance, CA, USA). The organic acids were eluted isocratic with 5 mM sulfuric acid (H2SO4) with a flow rate of 0.60 ml/min. The column oven was held at a temperature of 60°C. Data analysis was performed with Chromeleon software v.7.2 (Thermo Fisher Scientific). The result was calculated using two concentrations of a standard mixture containing all relevant organic acids, which was used as a reference sample in each continuous series of analysis

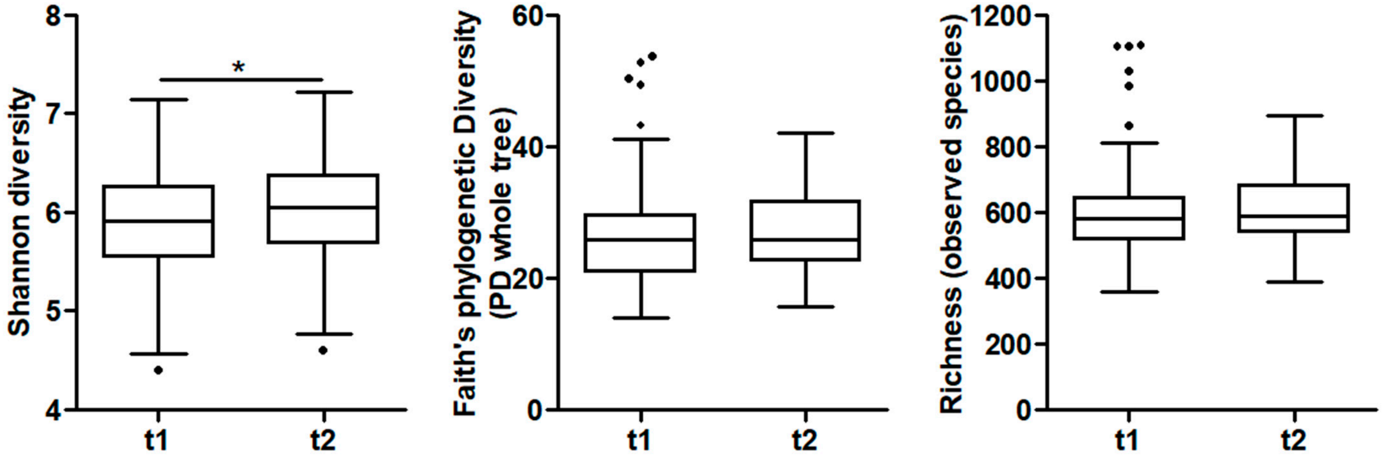

Figure S1. Shannon diversity index (left) was slightly higher at t2 compared to t1 (*p*=0.0176). Faith's phylogenetic diversity (middle) and Richness (right) were not different. Boxplots are displayed as Tukey whiskers.

Table S2 Taxa that were significantly different between t1 and t2 in bivariate analysis of each taxon, after correction for multiple testing by FDR. The mean 2log ratio column is conditionally formatted, in which an increase is indicated by positive (red) ratios, and a decrease by negative (blue) ratios.

Phylogenetic level	Taxon	t1 mean relative abundance (%)	t2 mean relative abundance (%)	Mean 2log ratio	p-value	p-value FDR
family	Clostridiaceae	5.44	3.72	-0.55	1.67E-06	4.03E-04
family	Ruminococcaceae	17.90	20.48	0.19	7.74E-04	1.56E-02
genus	Coprococcus	7.38	8.09	0.13	2.07E-03	3.26E-02
class	Bacilli	2.34	1.34	-0.80	2.25E-05	1.46E-03
order	Lactobacillales	2.11	1.19	-0.82	5.58E-05	2.75E-03
family	Streptococcaceae	1.74	0.88	-0.99	7.74E-06	7.62E-04
genus	Streptococcus	1.69	0.87	-0.96	2.59E-05	1.46E-03
genus	Lactococcus	0.06	0.01	-2.47	1.15E-05	9.05E-04
phylum	Bacteroidetes	3.08	4.12	0.42	8.70E-04	1.56E-02
class	Bacteroidia	3.08	4.12	0.42	8.70E-04	1.56E-02
order	Bacteroidales	3.08	4.12	0.42	8.70E-04	1.56E-02
family	Bacteroidaceae	1.38	1.88	0.45	6.21E-04	1.44E-02
genus	Bacteroides	1.38	1.88	0.45	6.21E-04	1.44E-02
family	Rikenellaceae	0.32	0.45	0.50	2.78E-04	1.20E-02
family	[Odoribacteraceae]	0.05	0.08	0.81	2.05E-06	4.03E-04
genus	Butyricimonas	0.02	0.03	0.77	5.03E-04	1.32E-02
genus	Odoribacter	0.03	0.05	0.85	3.44E-06	4.51E-04
family	Alcaligenaceae	0.02	0.04	0.64	3.90E-04	1.20E-02
genus	Sutterella	0.02	0.03	0.62	3.96E-04	1.20E-02
class	Deltaproteobacteria	0.02	0.03	0.85	3.35E-03	4.98E-02
order	Desulfovibrionales	0.02	0.03	0.85	3.54E-03	4.98E-02
family	Desulfovibrionaceae	0.02	0.03	0.85	3.54E-03	4.98E-02
order	Actinomycetales	0.09	0.06	-0.42	3.84E-04	1.20E-02
family	Actinomycetaceae	0.07	0.05	-0.31	3.56E-04	1.20E-02
genus	Actinomyces	0.06	0.05	-0.31	6.89E-04	1.51E-02

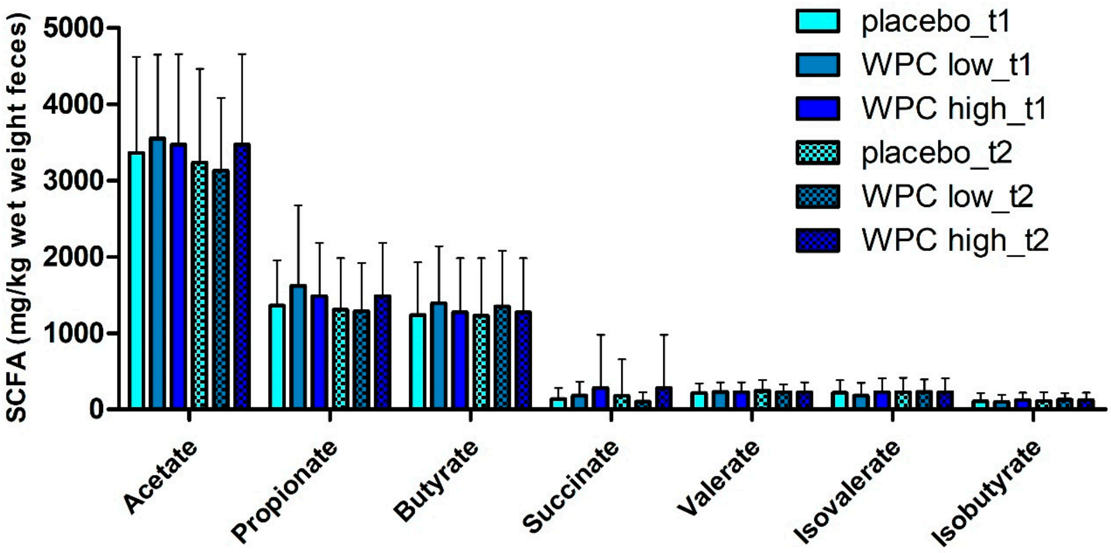

Figure S2. Fecal SCFA (mg/kg wet weight feces) as determined by HPLC. There were no statistically significant differences between the treatment groups.
